# Yarning as decolonial praxis in initial teacher training: an Australian context

**DOI:** 10.3389/fsoc.2025.1561532

**Published:** 2025-07-02

**Authors:** Aleryk Fricker

**Affiliations:** Deakin University, Waurn Ponds, VIC, Australia

**Keywords:** Yarning circles, decolonisation and education, teacher education, Aboriginal, autoethnography

## Abstract

Yarning has been a widespread practice for First Nations people across the Australian continent for approximately 70,000 years. Yarning as a process of communication has been designed to support authentic and relational connections between people, Country, ancestors, spirits, and the more-than-human realms. In recent scholarship, the process of yarning has emerged in a western context as being a legitimate research method for gathering rich qualitative data. It has also been found to be able to support social connections, collaborations, and processing and sharing trauma. This paper explores collaborative yarning as a pedagogical process in initial teacher training in Australia through auto-ethnographic reflections, and how engaging with yarning as a pedagogical process can challenge the neo-colonial pedagogies that have dominated higher education in Australia for over a century. This paper has found that when engaging with yarning in Higher Education, it can provide an important opportunity to reduce the neo-colonial violence present.

## Introduction

Schooling as it is experienced in contemporary Australia is a colonial construct. Prior to the invasion, colonisation, and genocide in Australia beginning with Cook’s claim over the Australian continent and adjacent islands in 1770, young people had been successfully educated for millennia. Within two decades, this was disrupted further with the arrival of the First Fleet under Captain, and then Governor, Arthur Phillip in 1788. This arrival sparked the beginning of the continental genocide, erasure, and replacement of First Nations people and contexts, as part of the expansion of the British Empire (Wolfe, 2006).

As part of the colonisation of Australia, the newly arrived colonists sought to destroy and replace the systems and structures which had existed for up to 70,000 years (Wolfe, 2006). One such system usurped by the colonists was the education system. The new colonial system initially denied access to First Nations children with the exception of the Paramatta Institute that, for a short period from 1814 to 1833, focused on ‘civilizing’ the surviving children of the First Nations families around Sydney Town, but this was eventually abolished due to a lack of children attending (Robinson and Paten, 2008). The outcome was an education system that actively prevented First Nations children’s access for over a century, and one, when access was finally granted, that limited First Nations outcomes while actively seeking to, in some cases violently, assimilate them into a national colonial ideal (Burridge and Chodkiewicz, 2012).

The University of Sydney was first university established on the Australian continent in 1850. Shortly thereafter, in the immediate wake of the Victorian Goldrush, the University of Melbourne was established in 1853. These institutions fulfilled an important role for the local colonist students. For many, they no longer needed to take the arduous journey to the British Isles to access Higher Education, and could access degree level qualifications in a local context.

The establishment of the western and British style of education across the newly established British colonies effectively overwrote the education systems that had been well established across the Australian continent and adjacent islands for millennia. The outcome was an education system that was not culturally responsive, and one that sought to perpetuate settler-colonial futurity through the replication of colonial pedagogies, curricula, systems, and policies that were often hierarchical, adversarial, and competitive (Rudolph, 2023).

The result was a schooling system that focused on the outcomes of European students with little thought or access to education given to First Nations children. This was also a system that sought to recreate the European onto-epistemologies, which framed all aspects of the schooling’s teaching and learning experience, and actively perpetuated carceral logics (Rudolph, 2023), colonial mythologies (Stanner, 1969), and settler futurity (Belcher, 2024) across the newly colonised lands.

## Context schooling in Australia

Over the course of two centuries, the legal fiction of Terra Nullius was declared across the continent and the adjacent islands to justify the dispossession of Country from First Nations Peoples, and an erasure and denial of the existence of First Nations sovereignty (Buchan and Heath, 2006). This remained until it was overturned during the landmark Mabo decision in the Australian High Court in 1992.

With the arrival of the British, the invasion, colonisation, and genocide began in earnest, and colonial systems were established to benefit the so-called ‘Free Settlers’; these remain in contemporary Australia. This included all systems of government and private service provision, which included education systems that replicated those in England from the 19th Century onwards. Initially, this was done as a form of social engineering to both gain benefit from the newly extracted riches from the recently stolen lands, as well as to benevolently provide a system of education that would be able to reform the children of the convicts transported to Australia (Campbell and Proctor, 2014).

Embedded within this newly established colonial schooling system were also Eurocentric onto-epistemologies, which shaped both the content within the curriculum and the preferred pedagogy used deliver this content. This meant that for the colonial children fortunate enough to be able to access this schooling system, they engaged with content familiar to them and their cultural contexts, taught in ways which also met their culturally shaped onto-epistemological expectations (Hickling-Hudson and Ahlquist, 2003).

The positioning of First Nations people as being unintelligent and a ‘missing link’ between humans and primates was a myth propagated well into the 20^th^ Century, where even globally respected science magazines continued to publish articles making these false claims. These were retracted in a special issue of National Geographic magazine in 2018, which focused exclusively on race and apologised for the harm caused in previous editions of the magazine where race was a focus (Goldberg, 2018). One article and image of two First Nations adults was highlighted as a significant error as the caption for the image originally published in 1916 claimed that Aboriginal people were ‘savages’ and ‘ranked lowest in intelligence of all human beings’ (Goldberg, 2018). This prominent and prevailing viewpoint was a key factor in the restriction of schooling that many First Nations children experienced right up to the early 1970s in Australia. Access to education for some First Nations students is still restricted, especially in very remote contexts (Fricker, 2021).

For First Nations children, with the exception of the Paramatta Institute, there were no efforts to provide any kind of state-based schooling experience, and what little was provided was often unfit for the students, their families, and often did not meet community expectations. In some isolated instances young First Nations children would be taught under the guise of what would be considered private tutoring, but for the majority, attempting to educate a First Nations child was considered to be a waste as it was commonly thought that First Nations people were ‘savages’ and incapable of intelligent or complex thought (Burridge and Chodkiewicz, 2012).

Over the next few decades, the initial exclusion policy by which First Nations children were prevented from accessing schooling shifted to one where First Nations children could begin to access public schooling. This access, however, was tenuous at best, with many schools adhering to a policy whereby any First Nations child could be excluded from the school at the request of a non-Indigenous parent (Cadzow, 2007). For the most part, experiences of schooling during this period were in segregated mission or reserve schools, or in segregated classes in public schools (Burridge and Chodkiewicz, 2012). This was further impacted by an expansion of the powers of the Protector of Aborigines who, in the early 20th Century, was given the authority to remove any Aboriginal child considered to be ‘neglected’ from a schooling context. This meant that there were many Aboriginal children who attended school 1 day and did not return home afterwards (Burridge and Chodkiewicz, 2012). These children ultimately became part of the Stolen Generations.

By the 1920s, and in the face of a growing number of children being taken by the state governments across Australia, the first Aboriginal political association was formed to directly challenge the power of the Protector of Aborigines. This organisation was known as the Australian Aboriginal Progressive Association and was established by Fred Maynard and several other prominent Aboriginal leaders. One of its many policy focuses was on the abolition of segregated schooling for First Nations children (Maynard, 1997).

As the colonisation of Australia continued into the 20th Century, discrete policy positions of assimilation, integration, and self-determination were adopted by the federal government (Short, 2003). The result was that the various federal, state, and territory education policies also began to shift to slowly allow access to higher schooling levels to First Nations students (McConaghy, 2000; Hogarth, 2016). Despite the access to school improving, First Nations students remained a segment of the student population that did not achieve at the same levels as their non-Indigenous peers (Hogarth, 2016).

A response to this was to establish policies which allowed the various state and territory based initiatives to employ First Nations education support staff variously known as Koorie Engagement Support Officers (Victoria), Aboriginal and Islander Education Officers (Western Australia), Community Education Counsellors (Queensland), Aboriginal Community Education Officers (South Australia) and Aboriginal Education Officers (New South Wales). First Nations student outcomes have remained below those of their non-Indigenous peers, but these staff being part of the front line of the implementation of support services to the First Nations students and families have also contributed to the shaping of contemporary Indigenous education policies in their respective jurisdictions (MacGill, 2017).

The overall outcome of these various education policies and the associated restrictions to the access of education was that the articulation of all First Nations students, through and beyond the schooling system was severely disrupted. This meant that despite many examples of First Nations children like Maria Lock in 1819 out-performing their non-Indigenous peers (Brook, 2008), no First Nations students were permitted to access and attend tertiary education for over a century after the first university was established in Australia.

The lack of engagement with Higher Education in Australia for First Nations students meant that the neo-colonial constructions, which existed within had few academics present, who had the knowledge or the inclination to challenge the erasure and subsequent positioning of First Nations knowledges. This was highlighted in 1961 when the Australian anthropologist W. E. H. Stanner was appointed to convene the first conference on the state of Aboriginal studies in Australia, and only non-Indigenous delegates attended (Moreton-Robinson, 2023). Despite several influential academics challenging the academy to make changes to support the inclusion of Indigenous scholars and scholarship, there still remained a desire for First Nations people to be constructed through a neo-colonial lens (Judd, 2014).

This absence also informed the pedagogical approaches used in Higher Education, where there was a faithful recreation of the teaching and learning techniques traditionally used in Europe, which the newly constructed Higher Education sector sought to recreate (Lake et al., 2022). These were heavily influenced by the Classical Greek period over 2000 years ago, where the Socratic Method provided a pedagogical framework to support students engaging with the content they were learning, as well as shaping their interactions with their peers and the academics (Detweiler, 2022).

The Medieval Period in Europe, as the time when the first European universities were established, also had a significant influence on the establishment, implementation, and expectations of what the pedagogies in the classroom would look and feel like. This process was shaped through the establishment and continuation of the medieval trivium, as well as the pedagogies being shaped by the common experiences and fears of violence occurring in the same places as the newly established universities (Enders, 1999).

The outcome has been one where there has been a tension built into the pedagogical experiences between teachers and students (Detweiler, 2022) where violence has been embedded as part of the pedagogical experiences (Enders, 1999). This means that it has not been uncommon for students to experience Higher Education which included significant distance between the teacher and student (Anderson, 1979), and that this distance can promote poor behaviour in the classroom also (Boice, 1996).

Additionally, when the student relationship is strong, it can reduce rates of student absenteeism (Rocca, 2004), as well improving the efficacy of affective learning for students (Witt et al., 2004). This all has an impact on student experience and academic results.

In the Australian context, Higher Education as a colonial construct has also included the construction of specific Eurocentric pedagogies, which have been influenced by European classical and medieval onto-epistemologies, which in turn, have sought to establish tension, violence, and distance between the teacher and student. This has led to teaching and learning experiences which are, at times, impersonal and lacking in any real relationship or relationality (Murphy and Brown, 2012).

## Contemporary Higher Education in Australia

Over the last few decades, there has been a steady increase in the awareness and engagement with First Nations contexts in Higher Education. This has been marked by an increase in the number of universities engaging with Reconciliation Action Plans or Indigenous Strategies. Beyond the focus on research and the general university business, another area that universities have been actively seeking to engage with is First Nations content and pedagogies across many different discipline areas (Marsh et al., 2023).

There have been some tensions arising from this work, with universities seeking to extract First Nations knowledges with a view to improving student and research outcomes, but without seeking to engage with the work of decolonial praxis that would enable a greater and more in-depth engagement with the First Nations contexts. An outcome of this has been that, despite an overall increase in the numbers of First Nations students completing tertiary qualifications during the last few decades, universities have had much lower employment of First Nations academics; well below what would be expected for population parity (Wilson and Wilks, 2015).

There is some will in the sector to generate reform to improve the engagement with First Nations contexts however, overall, the sector is still dominated by non-Indigenous pedagogical traditions, which shape both the learning and teaching experiences of students, and the ways that knowledges are positioned within the academy. This has been a contributing factor for poor experiences for First Nations students (Sonn et al., 2000).

This has been further complicated by an ongoing focus and tension within Teacher Education programmes across the country being blamed for poor student outcomes, and in some ways the ongoing impact of the legacy of the historical association with teaching colleges established initially as separate entities from Higher education (Aspland, 2006). The tensions within this aspect of teacher education comes from the context where training to become a teacher was initially structured as an apprenticeship model where the learning largely occurred on the job. In the 1980’s teacher colleges around Australia were dismantled or absorbed into universities and a more scholarly approach was embraced. One legacy has been that conservative political ideologies in the Australian political system have increasingly been agitating to move towards a deprofessionalised teacher education model much closer to the original apprenticeship style (Buchanan, 2020).

As argued below, in the context of a neo-colonial Higher Education sector in Australia, a key strategy to improve outcomes for all students would be to decolonise the pedagogical approach in order for it to be Indigenised. This would not only support the engagement and outcomes of First Nations students, but also provide positive outcomes for all students, regardless of their cultural contexts.

## Decolonising pedagogy for Indigenous contexts

When considering the process and application of decolonial praxis in Higher Education, the models of Fricker and Fricker (2023), and Hughes and Fricker (2024) have been used to shape this approach. The Fricker and Fricker (2023) model has identified five focus areas to consider in the classroom when working to apply decolonial praxis. These include curriculum, place and space, community engagement, policy, and pedagogy. They argue that these five aspects, as experienced in the contemporary Australian education system, are colonial constructs and as such, it is imperative that they are dismantled and reimagined through a lens of decolonisation. They also argue that this should be the work of all teachers, regardless of their own cultural contexts (Fricker and Fricker, 2023).

This model is further reinforced by Hughes and Fricker (2024), where they argue that the division of labour as part of a process of decolonial praxis must be split between Indigenous and non-Indigenous stakeholders. This is to ensure that the colonial load is better managed for Indigenous stakeholders, as well as ensuring that appropriate leadership and oversight is maintained so that unceded Indigenous sovereignty can be manifested and realised through the process.

The focus on decolonising also provides an important opportunity to include a culturally responsive pedagogy (Cumming-Potvin et al., 2022) which better enables the manifestation of Indigenous ways of experiencing learning and teaching, which are often centred on relationships and relationality (Tynan, 2021). As argued above, these are diminished within the space of the neo-colonial pedagogical context in Higher Education, which is dominated by the previously mentioned tension, distance, and violence.

There is a growing number of Indigenous pedagogies being experienced throughout the Higher Education system. These range from taking students out of the university classroom and having them learning directly from Country and Elders. This is known as On Country Learning (Moran et al., 2018). Another is known as the 8-Ways, which is a pedagogical model that blends many different learning and teaching approaches within a holistic framework to support the academic and well-being outcomes for students (Yunkoporta, 2009).

## Yarning as decolonial pedagogy

The decolonial pedagogical approach used in this paper is Yarning. This is an approach which has had multiple applications in a research method and methodology context (Bessarab and Ng'andu, 2010; Geia et al., 2013; Dean, 2010), but has so far had limited exploration through research as a pedagogical approach (Brigden et al., 2020) despite having a growing presence in the contemporary Australian classroom, and multiple government department of education resources to support its implementation (Queensland Curriculum and Assessment Authority, 2025).

Yarning as a pedagogical approach has been described as a slow pedagogy (Brigden et al., 2020) that allows all students ample time to engage with the learning in the classroom. It also has implications for reducing the hierarchical structures present in Higher Education classrooms and through encouraging relationships and relationality between students, students and instructors, and students and the content they are learning, yarning as a pedagogical technique has begun the work of challenging neo-colonialism present in the Higher Education classroom.

## Auto-ethnographic method

Autoethnography has been used in this paper as the method of gathering the data for this study through its suitability to be used in the context of decolonisation. As a method, it provides advantages through being focused on challenging canonical ways of conducting research as well as treating research as a political, socially conscious and a just act (Ellis et al., 2010). This method is characterised by an understanding that it can be considered in two broad categories. The first is autoethnography as process, where the author uses hindsight to document experiences and reflections of past events (Freeman, 2004). This method is useful as it has allowed the author to both recall events and reflect on their meanings, contexts, and in some cases, just like a yarn, meanderings.

The second characteristic is autoethnography as a product. This aspect considers the output of the study, which is often punctuated to bring readers ‘into the scene’; specifically, into thoughts, feelings, and actions (Ellis, 2004, p. 142). This approach also allows any author to construct a ‘thick description’ of cultural practices, which can effectively facilitate understandings of culture for both insiders and outsiders which, in turn, increases the accessibility of the scholarship (Goodall, 2001).

Autoethnography also allows for a disruption of western hegemonic onto-epistemological dominion via the opportunity to construct Indigenous research data through authentic Indigenous standpoints to connect the data to larger historical, cultural, social, and political realities (Tamarapa, 2024). It also emphasises the role of researcher and the participant and narrator, and acknowledges the interplay between the *personal* and the *collective* in knowledge construction (Lamichhane and Luitel, 2023).

Finally, this approach also supports my own cultural contexts, which require the centring of storytelling as a foundational cultural practice required to honour and maintain the cultural continuity of the way knowledge has been shared on Country for millennia, and will continue into the Dreaming (Heaslip Kefi, 2023).

## Case study

This section of the paper explores reflections through a vignette of my own engagement with Yarning Circles as a specific pedagogical process. By facilitating yarning circles, I have been able to implement important pedagogical processes into my Initial Teacher Training units and have provided training to my students to enable them to deliver yarning as a pedagogical approach in their teaching contexts also.

## Vignette

Before every yarn I get both nervous and excited. It is like the ultimate existential experience, where no amount of planning or consideration can predict where it will go, how it will be experienced, or any of the possible outcomes. Despite the nerves and excitement, it is also paradoxical as it is one of the most comfortable things to do in the world. It is something that has always been present in my life. For as long as I can remember, I have been yarning with others, albeit not having the reflective capacity or the language to articulate it, but nonetheless, it has been present in my life.

Some of my early interactions with yarning were not positive. Growing up in a largely non-Indigenous and homogeneously white outer suburb of a major city in Australia meant that, when I would seek to yarn with my classmates, expecting some sort of reciprocity, I would often be labelled as an ‘over sharer’ or someone who could not get to the point. The result would be that the stories about myself that I shared would be weaponised against me and would come back in negative ways. I learned very quickly that the non-Indigenous students I was learning with both could not yarn, and did not value it enough to want to try to learn.

As such, I was given a lifetime of experiences to learn that most non-Indigenous people cannot yarn and many, when confronted with yarning by another person, do not actually know what to do, or how to respond. This is my starting point with my yarning circles as part of the decolonising of pedagogy in my Indigenous Education unit within the initial teacher training programmes.

This means that when I yarn with students, I not only have the yarn itself going, but I also have a meta-yarn, which I use to pause, explain, and make visible, the various aspects of the yarn, the rationale behind them and discussions about how we are all feeling and why. This means that I begin the yarn with a set of expectations. I choose not to use the term ‘rules’, as this, I feel, has an impersonal emphasis, which is at odds with what a yarn is supposed to achieve and reflect.

The first expectation is that the content of the yarn can and should only be shared with those who were a part of the process. This is designed to protect the integrity of the trust, understanding, solidarity, and community, which is fostered within the yarning circle. This makes it clear to the participants that any sharing or weaponisation of the vulnerabilities shared within the yarn will not be tolerated.

The next expectation is that all participants and their contributions to the yarn will be honoured by all. As we share our stories, insights, reflections, and intentions within the yarn, we are giving each other gifts. These should be honoured at all times, which means that we engage one another with respect, empathy, and understanding, even on points of opposition or contention.

The next expectation is that the richness of the yarn is enhanced by the number of voices who choose to join it (the more the better). This is by no means an effort to coerce students to participate, but it is to make clear that space must be made for students who would not usually speak, as well as making it clear that we all have responsibility to enrich the learning experiences for each other, and this can only be done through generosity and engagement with the yarn.

The next expectation of the yarn is that students will prioritise their capacity to build and be in relationships with themselves, each other, and to the knowledge that they will share and receive. This encourages students to reflect deeply, think critically, and engage with the yarn with purpose and intent. It seeks to foster considerations that knowledge can and should be experienced as much more than the commodity that it is often positioned as in western contexts.

The final expectation of the yarn speaks more to priming students to possibly have a unique learning experience. That is, to expect at times to feel something in the yarn. This is part of the process often at odds with western pedagogical traditions, where efficiency is often prioritised above effectiveness in the teaching and learning cycle, and that emotion and affective learning is positioned to the margins of pedagogical value.

Once the expectations have been set. We move into the first phase of the yarning circle. In this phase, either the yarning object, or ball of yarn will be passed around the circumference of the circle, and this is the only time in the yarn where participants are expected to speak. During this phase, the prompt accompanying the object/ball of yarn is a low-stakes simple response for example: ‘What is your name? What are you studying? And if you could be any other animal, what would you be and why?’

This is often the first point where I will pause the yarn and discuss the meta yarn aspect. In this case, I make it clear that I will go first with each of the provocations or phases of the yarn, as this is about modelling potential answers for the students so they might observe and consider their own answers. This is especially important in contexts where participants have not yarned before. This also provides another very important role in that, as I model my answers as the facilitator of the yarn, and often the assessor of the students, I have an opportunity to make myself vulnerable through the stories I share. This is vital to ensure that the participants begin to feel safe to share their stories, as well as dismantling the neo-colonial power structures present within the Higher Education classroom.

A point that I also make at this stage of the yarn is the importance of the ball of yarn, if we are using one. This object not only provides the right to speak, but as the ball of yarn moves between people in the circle, it is unravelled to ensure that the yarn begins to create a pattern within the circle. At the conclusion of the yarn, I like to use it as a visual metaphor for the messiness of learning, relationships, and knowledge, which is often ignored through a western pedagogical lens of efficiencies.

As students around the circle provide their answers to the initial low-stakes prompt, they are often met with some chuckles and positive responses from their peers. It is not uncommon for students to desire to be the same animal as an earlier speaker, and to provide the same reason. At this point, the body language of the group becomes more comfortable as they begin to connect and consider each other’s responses.

Once the object/ball of yarn has been returned to me, we usually begin the next prompt or provocation. At this point I pause the yarn again and describe how it will now change. At this stage, the object/ball of yarn will now be passed across the circle, rather than around the circumference. In the case of a ball of yarn, it will often be thrown across the circle; in the case of an object, it is often not appropriate to throw it and rather, it will remain in the centre unless a participant leaves their seat to pick it up, return to their seat and then speak before returning it once they have finished. I have found that this structure tends to work well with participants who have had some practice, as inexperience often comes with anxiety that we will run out of time, and this has resulted in multiple participants grasping for the object at the same time.

With this phase of the yarn, I will also mention that no one is obliged to speak unless they wish to, but remind the participants that the richness and the learning of the yarn is dependent on the number of people who decide to speak. I then begin by providing the next provocation. This relates to the importance of Country for First Nations people and the provocation is: ‘Where is your special place? Why is it special to you?’ In this case, I do not provide any examples i.e.: Home, the beach, holiday house, for example, as I do not want to unduly influence the participants. Rather, I provide a story about my special place and speak to a time when my Dja Dja Wurrung grandfather passed away from a brain tumour and prior to his death was called home to Country. What provides some of the power of this story is that my grandfather did not identify as First Nations and at that time we were only beginning to make the connections back to our ancestral homelands.

When I share this story I often get emotional and this, too, is an important and powerful gesture to build safety and connections with the participants. In these moments we are directly challenging the violence held within western pedagogies in Higher Education, as well as the power structures in the classrooms, which are perpetuated by these same violences.

Before I ask the participants to share their stories, I pause the yarn again to prepare them for what could happen next. I make it clear that I will thank each participant for their contribution (without myself holding the object/ball of yarn) to honour their contributions and wisdom. I also discuss the affective nature of the yarn and how it supports participants to learn through and with an emotional response. In this case, we want to feel our learning. I also make it clear that if anyone is distressed and needs to step away, they are welcome to without judgement. I also encourage participants to show their humanity and, with consent, to comfort anyone experiencing a strong emotional response. This can be a simple as passing a tissue, or in some cases a warm embrace; responses not typical of a higher education classroom.

As the yarn moves between the participants, we often get a range of responses. For some, there will be required some more experience with this process to build enough trust and comfort to share vulnerable information about themselves. For others, this provides an important opportunity to share parts of themselves, which are important and authentic. During this provocation, there are often common responses, which often involve being close to water at the beach, the ocean, or specific freshwater places around the world. For others, the physical place is not so important, but rather the people who are also present there are. Some of the less common responses have been participants who have mentioned virtual places and others who have mentioned specific rooms in specific homes as well.

During this provocation, it is not uncommon to have many moments of silence when no one is speaking. During these times of reflection and contemplation, I will occasionally pause the yarn and speak about the phenomenon of thinking time and the importance of letting students think about their responses. I will also often discuss the importance of allowing participants to engage on their terms without the violence of coercion.

At the conclusion of this provocation, time permitting, I’ll finalise it with a third provocation, where I get the participants to make comparisons between this way of learning and the ways that they are most familiar with. The results are often enlightening as they share how they have been impacted by the process of yarning. A common response is that they feel more connected to their peers in the yarn, as well as the knowledge itself. There are often comments, which highlight the emotional impact of the yarn and one where participants have remarked that fearing emotions in the classroom is often an unfounded consideration.

To complete the yarn, I finish it by speaking about First Nations artworks, many which, in the tradition of the sand drawings of the Central Desert region, or the bark paintings of Far North Australia, can be ephemeral in their existence. In this case, I gather the tangled yarn and comment that even though the visual representation has been gathered, the knowledge and the feelings we have experienced will remain and we are forever changed.

Consistently, students will comment about this experience in the student feedback questionnaires as being one of the highlights of the unit, and mention that they are grateful that they feel confident and competent enough to begin to implement these processes in the classroom. I have others who comment on the importance of the pace of the yarn, and the learning not feeling hurried. Others comment about the un- or semi-structured experience of the learning, and still others comment on how effective learning through and with emotion was impactful on their own experiences, and the connections to their peers, which they otherwise feel they miss out on in other classes that implement neo-colonial western pedagogies.

## Outcomes

The outcomes of engaging with yarning as a pedagogical process in the initial teacher classroom cannot be understated. From my own experiences, I have found that by implementing yarning in the sessions, I am able to provide students with experience and confidence to begin to decolonise their pedagogical approaches for the benefit of all of their students, regardless of the cultural contexts present. From interactions with students, I have found that, after the sessions where we yarn and as we revisit this approach over the course of several sessions, the student engagement and interactions become richer and we are able to transition into a space where we have collectively become a learning community through building relationships and relationality.

In terms of the content covered in the unit I teach, this process also supports the content focused on First Nations onto-epistemologies. By implementing yarning where relationships and relationality are prioritised, I am able to model with my students how the First Nations onto-epistemologies around knowledge, and teaching and learning are constructed, co-constructed, and reconstructed to highlight how pedagogies are cultural constructs, which shape and are in turn, shaped by knowledge, and learning and teaching expectations.

From a personal perspective, this also provides me with opportunities to engage with my students in ways which honour my own cultural contexts. As a Dja Dja Wurrung academic, there are ways that I engage with knowledge, and teaching and learning, which ‘make sense’ to me, and I am then able to introduce them to my students. The outcome is also that I am able to reduce the amount of neo-colonial pedagogical violence, which is often present in the Higher Education context in Australia, and am able to model ways to engage with learning, which both challenge and complement western pedagogical approaches.

## Conclusion

The Higher Education sector in Australia is a colonial construct in need of significant reform. One of the ways of achieving this is through a focus on decolonial praxis in Higher Education in which pedagogical practice is one important focus area (Fricker and Fricker, 2023), and must be considered as part of the overall project to better embed First Nations contexts across this sector (Hughes and Fricker, 2024).

For many students who engage with the mainstream Higher Education sector in Australia, there is violence and tensions present, and a way of reducing the negative impacts of these phenomena is to construct learning communities, which can be supported through yarning. By incorporating yarning into everyday practice in universities, we are better able to challenge the neo-colonial hierarchy present within the classroom and enhance peer to peer learning.

To be able to learn through the affective domain is also another advantage of this approach in the classroom, and this, too, challenges a neo-colonial onto-epistemology, which considers good learning as being learning in which emotion is absent. Finally, there are few things more pleasant than being able to build collaborative, supportive, empathetic and effective relationships with my students, where I, too, get the benefit of getting to know them and building positive relationships with them.

In many ways, the contemporary education system in Australia has a lot to learn from First Nations ways of teaching and learning. As a member and beneficiary of the oldest continuous cultures in the world, every day I benefit from the oldest teaching knowledges and pedagogies in the world, and I often wonder, why we would not want all of these knowledges to be shared with all of our students, so everyone can benefit as I have.

## Data Availability

The datasets presented in this article are not readily available because this is an auto-ethnographic study as such, the data generated is present within the article. Requests to access the datasets should be directed to al.fricker@deakin.edu.au.
